# In Situ Raman Study of Neurodegenerated Human Neuroblastoma Cells Exposed to Outer-Membrane Vesicles Isolated from *Porphyromonas gingivalis*

**DOI:** 10.3390/ijms241713351

**Published:** 2023-08-28

**Authors:** Giuseppe Pezzotti, Tetsuya Adachi, Hayata Imamura, Davide Redolfi Bristol, Keiji Adachi, Toshiro Yamamoto, Narisato Kanamura, Elia Marin, Wenliang Zhu, Toshihisa Kawai, Osam Mazda, Toru Kariu, Tomonori Waku, Frank C. Nichols, Pietro Riello, Flavio Rizzolio, Tania Limongi, Kazu Okuma

**Affiliations:** 1 Ceramic Physics Laboratory, Kyoto Institute of Technology, Sakyo-ku, Matsugasaki, Kyoto 606-8585, Japan; hyt8888@outlook.jp (H.I.);; 2 Department of Immunology, Graduate School of Medical Science, Kyoto Prefectural University of Medicine, Kamigyo-ku, Kyoto 602-8566, Japan; t-adachi@koto.kpu-m.ac.jp (T.A.); mazda@koto.kpu-m.ac.jp (O.M.); 3 Department of Dental Medicine, Graduate School of Medical Science, Kyoto Prefectural University of Medicine, Kamigyo-ku, Kyoto 602-8566, Japan; keiji922@koto.kpu-m.ac.jp (K.A.); yamamoto@koto.kpu-m.ac.jp (T.Y.); kanamura@koto.kpu-m.ac.jp (N.K.); 4 Department of Orthopedic Surgery, Tokyo Medical University, 6-7-1 Nishi-Shinjuku, Shinjuku-ku, Tokyo 160-0023, Japan; 5 Department of Applied Science and Technology, Politecnico di Torino, Corso Duca Degli Abruzzi 24, 10129 Torino, Italy; tania.limongi@polito.it; 6 Department of Molecular Science and Nanosystems, Ca’ Foscari University of Venice, Via Torino 155, 30172 Venice, Italy; riellop@unive.it (P.R.); flavio.rizzolio@unive.it (F.R.); 7 Department of Microbiology, School of Medicine, Kansai Medical University, 2-5-1 Shinmachi, Hirakata 573-1010, Japan; 8 Department of Oral Science and Translational Research, College of Dental Medicine, Nova Southeastern University, 3301 College Avenue, Fort Lauderdale, FL 33314, USA; tkawai@nova.edu; 9 Department of Life Science, Shokei University, Chuo-ku, Kuhonji, Kumamoto 862-8678, Japan; tkariu@shokei-gakuen.ac.jp; 10 Faculty of Molecular Chemistry and Engineering, Kyoto Institute of Technology, Sakyo-ku, Matsugasaki, Kyoto 606-8585, Japan; waku1214@kit.ac.jp; 11 Department of Oral Health and Diagnostic Sciences, School of Dental Medicine, University of Connecticut, 263 Farmington Avenue, Storrs, CT 06030, USA; nichols@uchc.edu

**Keywords:** outer-membrane vesicles, *Porphyromonas gingivalis*, Raman spectroscopy, phosphorylated dihydroceramides, cysteine proteases, amyloid β, hyperphosphorylated Tau

## Abstract

The aim of this study was to elucidate the chemistry of cellular degeneration in human neuroblastoma cells upon exposure to outer-membrane vesicles (OMVs) produced by *Porphyromonas gingivalis* (*Pg*) oral bacteria by monitoring their metabolomic evolution using in situ Raman spectroscopy. *Pg*-OMVs are a key factor in Alzheimer’s disease (AD) pathogenesis, as they act as efficient vectors for the delivery of toxins promoting neuronal damage. However, the chemical mechanisms underlying the direct impact of *Pg*-OMVs on cell metabolites at the molecular scale still remain conspicuously unclear. A widely used in vitro model employing neuroblastoma SH-SY5Y cells (a sub-line of the SK-N-SH cell line) was spectroscopically analyzed in situ before and 6 h after *Pg*-OMV contamination. Concurrently, Raman characterizations were also performed on isolated *Pg*-OMVs, which included phosphorylated dihydroceramide (PDHC) lipids and lipopolysaccharide (LPS), the latter in turn being contaminated with a highly pathogenic class of cysteine proteases, a key factor in neuronal cell degradation. Raman characterizations located lipopolysaccharide fingerprints in the vesicle structure and unveiled so far unproved aspects of the chemistry behind protein degradation induced by *Pg*-OMV contamination of SH-SY5Y cells. The observed alterations of cells’ Raman profiles were then discussed in view of key factors including the formation of amyloid β (Aβ) plaques and hyperphosphorylated Tau neurofibrillary tangles, and the formation of cholesterol agglomerates that exacerbate AD pathologies.

## 1. Introduction

Several phenomenological studies have associated chronic periodontitis with adult cognitive impairment [1,2,3,4,5,6]. These studies triggered further research focusing on the link between the infectious activity of *Porphyromonas gingivalis* (*Pg*) and Alzheimer’s disease, in search of possible mechanisms underlying the phenomenological findings [7,8,9,10,11]. *Pg* has long been known as a key oral pathogen causing microbial and immune dysbiosis [12]. In addition to possessing a stream of potent virulence factors, *Pg* exploits pathogenicity through its unique ability to subvert the host’s immune defenses [13,14]. Several *Pg* enzymes and proteins (both surface membrane and capsular ones) have been shown to be quite effective in suppressing the expression of neutrophil-recruiting chemokines, in cleaving the immune cell receptor, and in inducing subversive cross-talk signaling between toll-like and other innate immune receptors [15,16]. However, for the orally localized *Pg* infection to succeed in efficiently producing neurodegenerative processes from afar, one or more extracellular mechanisms are necessarily required [17]. In 2019, Dominy et al. [18] pointed to OMVs as representing the most lethal weapon that *Pg* possesses to initiate Alzheimer’s disease pathologies. *Pg* produces a unique class of cysteine proteases referred to as gingipains, whose delivery into cerebral microvascular endothelial cells occurs through *Pg*-OMVs (and not necessarily by direct infection) [19]. *Pg*-OMVs contain a high concentration of toxic virulence factors that are enveloped in a thin membrane that protects them until they can be delivered to tissue targets. In this way, toxins carried by *Pg*-OMVs can achieve swift long-distance transmission and amplify the pathogenic effects of *Pg* in the human body. It should be noted that OMVs are generally produced by several Gram-negative bacteria [20,21,22], but only those produced by *Pg* specifically contain the toxic virulence factors that initiate and progress Alzheimer’s disease pathologies [23,24,25].

In this study, we selected Raman spectroscopy as the primary analytical tool for separately characterizing the molecular components of *Pg*-OMVs, as well as monitoring physiological changes in neuroblastoma cells due to *Pg*-OMV-induced stress. In situ Raman spectroscopy is regarded here as a unique and powerful tool that is capable of providing new insights into the pathophysiological chemistry of AD, an urgent task in developing advanced diagnosis and preventive drugs. In our previous Raman studies of bacteria, fungi, viruses, cells and their metabolic changes [26,27,28,29,30,31,32], Raman spectroscopy was shown to be a uniquely suitable tool for pathogen identification, analysis of virus inactivation, and life-compatible metabolomic characterization on eukaryotic and prokaryotic cells. Specifically regarding extracellular vesicles, several research groups have performed Raman spectroscopic characterizations for those produced by eukaryotic cells [33,34,35], as well as prokaryotic ones, including *Pg*-OMVs [36]. However, there are currently no studies analyzing the Raman response in detail with the goal of interpreting the metabolic changes that *Pg*-OMVs induce when inoculated onto living neuroblastoma cells in comparison with a control sample.

Here, we build upon previously developed techniques for the extraction, purification, and activation of PDHC lipids and LPS [37,38], as well as the isolation and analysis of OMV phosphorylated ceramides [39,40,41,42], and delve further into the molecular-scale mechanisms that promote inflammatory responses in human neuroblastoma cells in vitro. The chemical signatures of in vitro SH-SY5Y living cells before and after exposure to *Pg*-OMVs were then corroborated with the results of transmission electron microscopy and fluorescence assays in order to gain internal consistency and to correctly assign molecular origins to the observed spectral variations. The present Raman characterizations also categorize and distinguish for the first time the spectroscopic response of PDHC and LPS in the context of the overall OMV structure, thus providing a list of key band assignments that could serve as a baseline in future analyses of the effects of abiotic stressors and drugs, as well as in clarifying previously unrecognized Pg-OMVs’ downregulatory roles in different autoimmune diseases.

## 2. Results

### 2.1. Transmission Electron Microscopy and Immunochemistry Assessments

**Figure 1:**
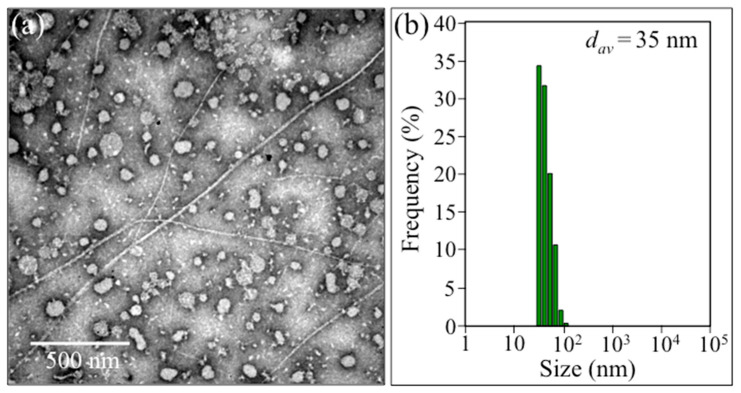
(**a**) TEM micrograph of Pg-OMVs and (**b**) their statistical size distribution as measured by DLS.

Figure 1a,b show the results of TEM observation and the statistical distribution of Pg-OMV size as measured by DLS, respectively. As can be seen, the morphology of the Pg-OMVs was nearly spherical, with an average diameter d_av_ = 35 nm. After adding the Pg-OMVs shown in Figure 1 (in a concentration of 10 μg/mL) to the SH-SY5Y cells and incubating for 6 h, the cell culture was observed with an optical microscope.

**Figure 2:**
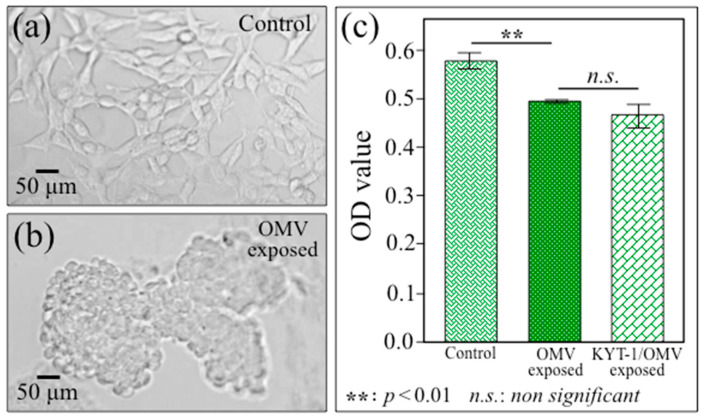
Optical micrographs of (**a**) unexposed SH-SY5Y cells (control) and (**b**) Pg-OMV-exposed SH-SY5Y cells; (**c**) results of OD cell viability assay on 6 h Pg-OMV-exposed cells in comparison with Pg-OMV-exposed cells in the presence of KYT inhibitor and unexposed control cells.

**Figure 3:**
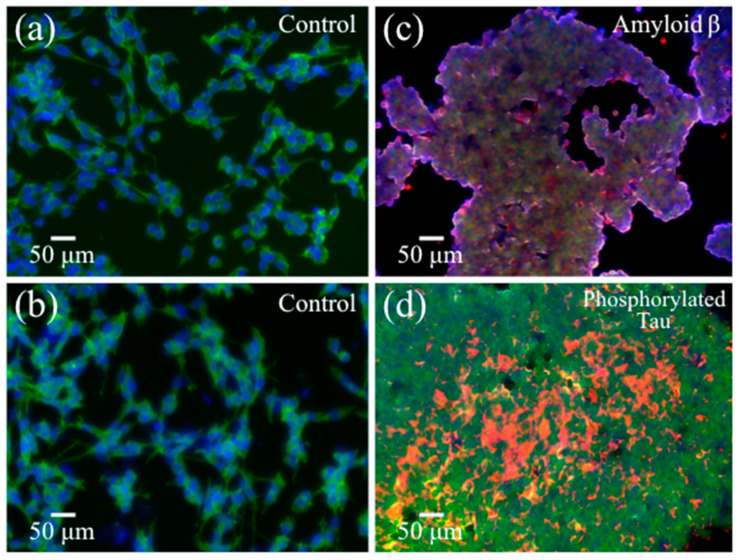
Fluorescence micrographs of (**a**,**b**) control SH-SY5Y stained cell cultures, (**c**) the SH-SY5Y cell culture shown in (**a**) after exposure for 6 h to Pg-OMVs (stained with Anti-Human Amyloid β (1-42) antibodies), and (**d**) the SH-SY5Y cell culture shown in (**b**) after exposure for 6 h to Pg-OMVs (stained with Anti-Phosphorylated Tau).

Figure 2a,b show optical micrographs of unexposed (control) and Pg-OMV-exposed SH-SY5Y cells, respectively. As seen, upon only 6 h exposure to Pg-OMVs, clear morphological differences could be observed between the two samples. Pg-OMV addition induced significant cell agglomeration with a tendency for individual cells to take on a roundish configuration. The cell viability of Pg-OMV-treated cells was also assessed on unexposed cells (control), cells exposed to OMVs, and cells exposed on OMVs treated with the gingipain inhibitor KYT-1 [43]. These latter results are shown in Figure 2c. The viability of SH-SY5Y cells was found to be reduced by ~14% compared to unexposed cells (control). However, despite having been reported as the most potent inhibitor of arginine- and lysine-gingipain (cysteine proteinases) [44], the KYT gingipain inhibitor did not reverse the cell neurodegenerative trend (cf. the statistically non-significant difference for cells treated in presence and absence of KYT in Figure 2c), thus suggesting that the activity of the OMVs is not due to gingipain only. Fluorescence microscopy was performed on cell cultures before and after exposure to OMVs. Control SH-SY5Y cell cultures were stained blue and green to visualize nuclei and actin filaments, respectively (Figure 3a,b). The SH-SY5Y cell cultures after exposure for 6 h to Pg-OMVs were stained with Anti-Human Amyloid β (1-42) antibodies (Figure 3c) and Anti-Phosphorylated Tau antibodies (Figure 3d). Fluorescence micrographs collected after staining with primary antibodies revealed dramatic structural changes and confirmed the above results, revealing the abundant presence of both Aβ and phosphorylated Tau peptides in Pg-OMV-exposed cells. The fluorescence microscopy data thus confirmed that formation of both Aβ and phosphorylated Tau occurred at an early stage as a consequence of contamination with Pg-OMVs and without the need for the direct involvement of Pg bacterial cells.

### 2.2. Raman Spectra of Pg-OMVs and Their Main Constituents

Phosphorylated dihydroceramide (PDHC) lipids can exist with three aliphatic chains (the substituted or “Sub” form; henceforth, SubPGDHC) or can be de-esterified to a lipid class with only two aliphatic chains (i.e., the unsubstituted or “Un” form; UnPGDHC). Pg is known to produce SubPGDHC copiously, but only a negligible amount of UnPGDHC [41].

**Figure 4:**
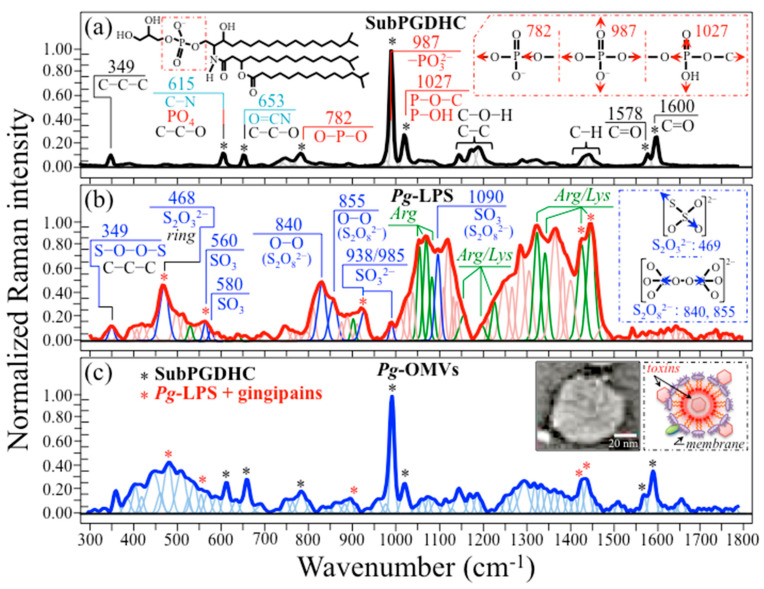
Average and deconvoluted Raman spectra in the wavenumber interval 300~1800 cm^−1^ collected for (**a**) SubPGDHC, (**b**) Pg-LPS, and (**c**) Pg-OMVs. Assignments for the main deconvoluted bands are provided. In the inset to (**a**), the molecular structure of SubPGDHC is depicted, while the insets in (**c**) show a high-resolution TEM image of an isolated Pg-OMV and a schematic draft of its structure. Red and black asterisks in (**c**) indicate bands mainly contributed by Pg-LPS (contaminated by gingipain toxins) and SubPGDHC, respectively. The abbreviations Arg and Lys refer to arginine and lysine amino acid residues, respectively, while the abbreviations C−C−C and ring refer to vibrational modes of glucose rings (cf. text). Red and blue arrows represent molecular vibrations for SubPGDHC and Pg-LPS respectively.

Figure 4a–c show Raman spectra in the wavenumber interval 300~1800 cm^−1^ as collected for SubPGDHC, Pg-LPS, and Pg-OMVs, respectively. The spectrum of SubPGDHC accurately reflects its chemical structure (in the inset to Figure 4a) by including C=O stretching (Amide I), C–H bending/scissoring, C–C stretching-related, and C–C–C deformation bands at around 1600/1578, 1450, 1150, and 349 cm^−1^, respectively, with all these signals arising from the three interconnected aliphatic chains [45,46,47]. Additional bands were found at ~653 and 615 cm^−1^, which represent O=C–N deformation (Amide IV) (with additional contribution from C–C–O deformation) and C–N deformation (secondary amide group, with additional contributions from C–C–O and PO_4_ deformations), respectively [45,48,49,50,51]. The strongest bands in the SubPGDHC spectrum (at 987 cm^−1^) and two weaker bands (at 782 and 1027 cm^−1^) were all related to symmetric or antisymmetric stretching of P–O bonds in the phosphorylated head group (cf. inset in Figure 4a). We also measured the Raman spectrum of de-esterified PGDHC lipids with only two aliphatic chains (UnPGDHC). The UnPGDHC spectrum was essentially the same as that for SubPGDHC, except for a lower intensity for the bands related to the aliphatic chains, namely, the C–H and C–C/C–O–H bands at around 1450 and 1150 cm^−1^, as could reasonably be expected due to their partially de-esterified structure.

Bacterial LPSs generally consist of three structural components: (i) lipids containing a hydrophobic (negatively charged) core, responsible for the toxicity properties [52]; (ii) a hydrophilic (positively charged) polysaccharide-chain core containing a short sugar chain as a connection between the lipid zone; and (iii) the so-called O-antigen, a repeating hydrophilic oligosaccharide side chain containing several different types of sugar [53,54,55]. The lipid hydrophobic core consists of a β-glucosamine-(1→6)-glucosamine-1-phosphate carbohydrate base attached to fatty acid esters. On the other hand, the hydrophilic polysaccharide-chain core consists of an inner part, which typically contains 3-deoxy-α-D-manno-octulosonic acid (KDO) molecules (attached to the disaccharide core), and glycan residues, which are typically phosphorylated and/or modified with phosphate-containing groups. The presence of phosphate groups in lipopolysaccharides increases the overall negative charge and helps to stabilize the overall LPS structure. The outer part of the lipopolysaccharide core includes common hexoses, such as glucose, galactose, and N-acetylglucosamine. The hydrophilic O-antigen consists of a repeating oligosaccharide unit that is distinctive to OMVs from each specific bacterium and possesses a high degree of structural variability. The significant heterogeneity of the LPS structure triggers a significant tendency for extrinsic molecules to be trapped, forming aggregates [56]. As a consequence of its complex structure, the Raman spectrum of Pg-LPS (Figure 4b) is quite rich in signals, which makes its interpretation quite challenging. As expected from the conspicuous presence of polysaccharides in its structure, the Pg-LPS spectrum included, among the most prominent signals, both glucose-ring-related bands at ~478 cm^−1^ and C–O–C bending signals at around 850 cm^−1^; additional signals with lower intensity were as follows: a composite signal of C–O and C–C stretching; C–OH bending at 1000~1150 cm^−1^; and C–H/C–H_2_ bending at 1300~1450 cm^−1^ [57]. However, the most prominent band at low wavenumbers was found at 468 cm^−1^, shifted ~10 cm^−1^ towards lower frequencies. This observation and the quite prominent nature of the Pg-LPS Raman bands in these two latter intervals suggest that the spectrum might contain a consistent number of signals from molecules different from carbohydrates, e.g., contaminations by gingipain toxins trapped in the lipopolysaccharide structure. Note also that glucosamine bands are expected to conspicuously contribute signals in the spectral zone 900~1500 cm^−1^; however, their strongest signals in this spectral zone, which are expected at ~865 and 1142 cm^−1^ (assigned to N–H and alkyl C–C stretching + ring breathing, respectively) [58], appear to be weakly represented in the spectrum of Pg-LPS. This observation strengthens the thesis that the unexpected population of strong signals resolved in the Pg-LPS spectrum is related to the presence of residual gingipain toxin contamination. Tentative assignments of the main observed bands are provided in Figure 4b. The bands from the contaminating molecules could be assigned to oxysulfur anions [59,60,61] and arginine [62,63] or lysine [63] residues (cf. labels in inset to Figure 4b). Further discussion on the origin of these bands will be provided in Section 4.1, below.

Figure 4c shows the Raman spectrum collected on isolated Pg-OMVs (cf. the enlarged TEM picture and schematic draft in inset). In the spectrum, black and red asterisks indicate the location of prominent bands for the SubPGDHC and Pg-LPS (+gingipains) samples, respectively. The strongest band in this spectrum appears at 987 cm^−1^. This band corresponds (together with the two companion bands at 782 and 1027 cm^−1^) to P–O bonds in the phosphorylated head group of SubPGDHC. Fingerprint signals for gingipain-contaminated Pg-LPS in the Pg-OMVs spectrum appeared at 469, 560, 938 cm^−1^ (i.e., bands contributed by oxysulfur anions) in addition to the arginine-/lysine-related signals at 1425 and 1449 cm^−1^ (CH_3_ symmetric and asymmetric bending, respectively) [62,63].

### 2.3. Raman Spectra of Amyloid β and Phosphorylated Tau Peptides

**Figure 5:**
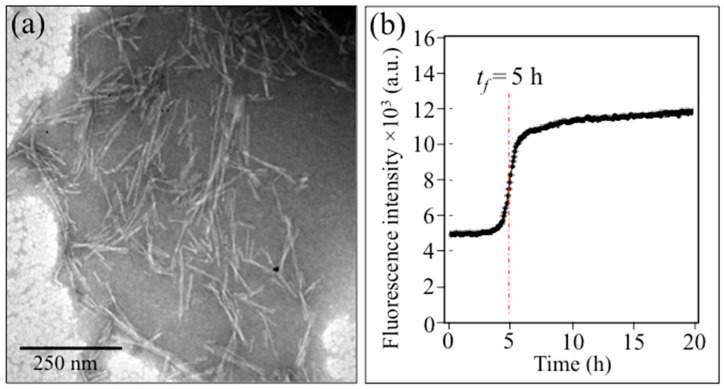
(**a**) TEM image of Aβ fibrils and (**b**) a plot of their characteristic fluorescence intensity upon staining as a function of time; the plot in (**b**) gives the kinetics of Aβ1-42 fibril formation as visualized by ThT binding assays.

Figure 5a,b show a TEM image of the investigated Aβ fibrils and a plot of their characteristic fluorescence intensity upon staining, respectively. The formation of Aβ1-42 fibrous aggregates was consistent with the findings of a previously published study [64]. The kinetics of Aβ1-42 fibril formation was investigated by ThT-binding assays. Since the quantum yield of ThT increases upon binding to the stacked β-sheets that form in the fibril core, ThT can be used to monitor Aβ1-42 fibril formation kinetics [65,66]. As shown in Figure 5b, the time course of ThT fluorescence intensity obeys a sigmoidal curve, which is characteristic of Aβ fibril formation [67].

**Figure 6:**
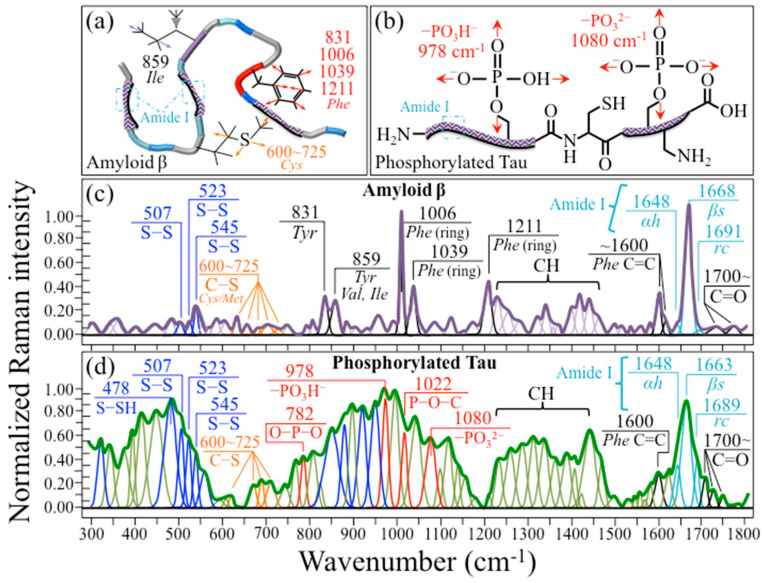
Schematic drafts of (**a**) an Aβ fibril and (**b**) a phosphorylated Tau filament; (**c**,**d**) their average and deconvoluted Raman spectra, respectively, as recorded in the wavenumber interval 300~1800 cm^−1^. Band assignments are given in the figure and described in the text. The abbreviations Cys, Met, Phe, Tyr, Val, and Ile represent cysteine, methionine, tyrosine, valine, and isoleucine amino acid residues, respectively. The abbreviations αh, βs, and rc represent α-helix, β-sheet, and random coil, respectively. Colored arrows represent the vibrations of the molecules in the respective insets.

In Figure 6a,b, schematic drafts are shown for an Aβ fibril and phosphorylated Tau filament, respectively, while Figure 6c,d present their respective average and deconvoluted Raman spectra in the wavenumber interval between 300 and 1800 cm^−1^. We decided to carry out preliminary Raman assessments of Aβ fibrils and phosphorylated Tau peptides in order to compare their mature structures with the actual structures formed upon cellular reaction to Pg-OMVs (cf. Section 2.4). These latter structures mainly include prefibrillar aggregate entities, referred to as oligomers, which have been reported to be equally toxic. According to Yoshiike et al. [68], the local structure of fibrils greatly affects surface properties, which are in turn responsible for the electrostatic and hydrophobic interactions with cells that lead to pathogenesis. Raman spectroscopy offers us a chance to characterize and discuss structure/surface property interactions.

The Aβ fibrils’ (average) spectrum in Figure 6c is dominated by ring signals from phenylalanine at 1006, 1039, 1211, and 1600 cm^−1^ (cf. the given vibrational assignments) [69], but, more importantly, it also includes signals of S–S and C–S bonds (from methionine and cysteine) at 500~550 and 600~725 cm^−1^, respectively [70,71]. Note that, unlike methionine, cysteine is not listed among the amino acid residues sequenced in Aβ fibrils [72]. However, we clearly detected (relatively weak) cysteine signals. Cysteine might be present in the tested Aβ protein structure as (external) surface groups. In addition, the S–S bands at 507, 523, and 545 cm^−1^ (cf. labels in inset to Figure 6c) have been reported to correspond to gauche–gauche–gauche, gauche–gauche–trans, and trans–gauche–trans conformations, respectively [73]. Unlike methionine, which is buried inside the protein structure, cysteine is exposed to the surface and presents a terminal thiol group, namely, a group consisting of a sulfur atom with two lone pairs, bonded to hydrogen. Disulfide linkages might contribute to the tertiary and quaternary structures of proteins, thus playing a crucial role in enhancing catalytic activity. Sulfhydryl groups are polar and hydrophilic in neutral aqueous solutions. However, in alkaline environments, they tend to form S–S bonds as disulfide links between cysteine residues, as schematically shown in Figure 6a. Molecular dynamics (MD) simulations of the aggregation of two insulin monomers in a β-sheet-rich dimer showed that dimers performed as templates for further protein aggregation, until the formation of fibrils [74]. This point will be discussed in further detail in Section 4.3, below. The tyrosine doublet located at 831 and 859 cm^−1^ represents an additional feature, although the latter band in the Aβ spectrum might also be contributed by C–C stretching in valine and isoleucine residues [75]. Regarding the information contained in the Raman spectrum about the secondary structure of the Aβ protein in the Amide I wavenumber interval 1600~1700 cm^−1^, a prominence of the band at 1668 cm^−1^ shows that the main (>90%) secondary structure is in the β-sheet configuration, while weak bands consisting of α-helices and random coils can be found at 1648 and 1691 cm^−1^, respectively. The recorded predominance of the β-sheet motif is in line with the findings of previous vibrational studies describing the secondary structures of the Aβ1-42 peptide and other Aβ alloforms [76,77].

Figure 6d shows the average Raman spectrum recorded on phosphorylated Tau protein in the wavenumber interval 300~1800 cm^−1^. The spectrum presents very intense bands arising from phosphate groups and disulfide bonds (cf. inset labels). The assignments of the two phosphate bands at 782 and 1022 cm^−1^ should be basically the same as those described in the previous section for similarly located bands in the SubPGDHC spectrum, namely, symmetric and anti-symmetric stretching of P–O bonds. However, Feng et al. [78] interpreted the band at ~1022 cm^−1^ as arising from a combination of the P–O–C and P–OH stretching modes of phosphate groups in hyperphosphorylated Tau (cf. inset labels). According to Xie et al. [79], the two bands seen at 1080 and 978 cm^−1^ can be attributed to protonated –OPO_3_^2−^ (dibasic) and –OPO_3_H^−^ (monobasic) phosphate structures, respectively. They showed that the Raman intensity ratio R_Ph_ = I_1080_/I_978_ could be used to perform local pH assessments using Raman spectroscopy. This point will be discussed in detail in Section 4.2.

The bands related to S–S bonds at 507, 523, and 545 cm^−1^ were the same as those observed in the Aβ spectrum (cf. Figure 6c,d). However, all S–S bands appeared to be relatively stronger, and with quite different relative intensities, than Aβ. In addition, a strong signal was detected at 478 cm^−1^. This signal was shifted by ~10 cm^−1^ towards higher wavenumbers compared to the band found in the Pg-LPS spectrum and assigned to S_2_O_3_^2−^ (cf. Figure 4b and Figure 6d). For this reason, we assigned this band, rather, to S–SH stretching [80,81,82]. The massive prominence of the 478 cm^−1^ band compared to the other S–S stretching triplet at 500~550 cm^−1^ suggests that the phosphorylated Tau structure abounds in hydrogen polysulfides. As discussed later, this spectroscopic detail is important and represents a well-known feature in the mediation of hypoxic regulation in cerebral microcirculation by the hydrogen sulfide pathway [83].

The secondary structure of the phosphorylated Tau protein, as detected from the Amide I vibrations at 1600~1700 cm^−1^, was found to be distinctly different from that observed in the Aβ spectrum (cf. Figure 6c,d). The most prominent band in this spectral zone was again that of the β-sheet (at 1663 cm^−1^), but bands of α-helices and random coils (at 1648 and 1689 cm^−1^, respectively) were markedly stronger than the corresponding ones in the Aβ spectrum. According to Avila et al. [84], Tau protein in solution is intrinsically disordered, and behaves as a kind of random coil, lacking a well-defined secondary structure. However, aggregation of such disordered proteins is associated with AD and other neurodegenerative disorders (i.e., the so-called tauopathies) [85]. Early studies by Kirschner et al. [86] and, later, by von Bergen et al. [87] showed evidence obtained by X-ray diffraction of β-sheet structures in Tau. Evidence for the presence of the α-helix motif in the Tau structure was first reported by Sadqi et al. [88] based on both circular dichroism and Fourier-transform infrared analyses. Subsequent studies confirmed this early finding on the basis of high-resolution NMR analysis [89,90]. The above studies suggested that α-helices could be found within the core of microtubule-binding domains and in the N- and C-terminal regions of Tau, an interpretation confirmed by Kunjithapatham et al. [91], who showed that the propensity of Tau to aggregate into α-helical structures was a consequence of its microtubule-binding capacity. The large number of phosphorylation sites present in the Tau structure [92,93] does indeed regulate the formation of tangles [93,94], with the phosphorylation process stabilizing the α-helix secondary structure [95]; the higher the content of α-helices, the higher the degree of hyperphosphorylation. The Raman data presented in this study are in good agreement with the above analyses of Tau secondary structures, revealing a prominent fraction of β-sheets together with clear bands of α-helices (stabilized by phosphorylation sites) and random coils. It is also shown here that Raman is capable of promptly resolving the secondary structure of Tau peptides despite their quite flexible structure and the large population of different conformations. Accordingly, Raman spectroscopy is confirmed here to be a suitable method for characterizing the Tau protein in AD pathology.

### 2.4. In Situ Raman Spectra and Imaging of Diseased SH-SY5Y Cells

**Figure 7:**
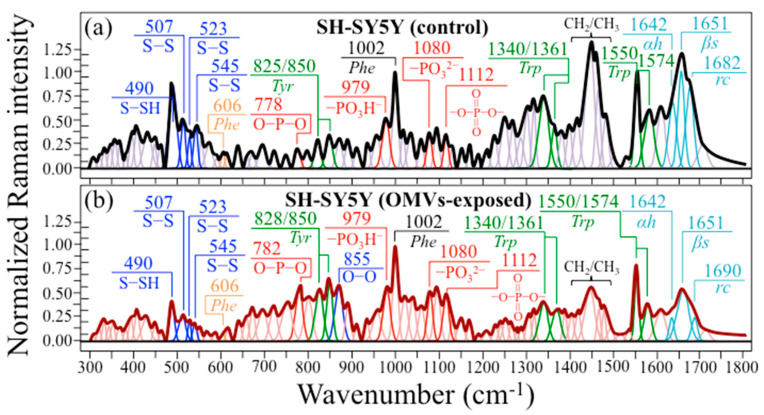
Averaged and deconvoluted Raman spectra in the wavenumber interval 300~1800 cm^−1^ as collected for (**a**) control SH-SY5Y cell culture and (**b**) OMV-exposed SH-SY5Y cell culture; band assignments are inset in the figure and described in the text. The abbreviation Trp represents tryptophan amino acid residues, while other abbreviations are the same as those in Figure 6.

Figure 7a,b show the average Raman spectra collected on control SH-SY5Y and 6 h OMV-exposed SH-SY5Y cell cultures, respectively. Both spectra were normalized to the ring-breathing phenylalanine band at 1002 cm^−1^ [69]. Despite the first-glance similarity between the two spectra, fundamental differences could be spotted upon accurate screening in different wavenumber regions. At lower wavenumbers, the S–S triplet at 507, 523, and 545 cm^−1^ and the S–SH band at 490 cm^−1^ can be observed in both spectra, as already discussed in the context of isolated Aβ and Tau spectra (cf. Figure 6). Despite possessing similar conformations within the triplet, a fundamental difference regarding the S–S-related bands resides in the ratio between the intensity of the hydrogen polysulfides signal at 490 cm^−1^ and the cumulative intensities of the disulfide triplet, which decreased ~2.5-fold in the OMV-exposed cells compared to the control. This bold difference in the population of sulfide bonds is a clear fingerprint of a depletion of “free-standing” (i.e., thiol-group-terminating) cysteine residues in the AD culture in favor of the formation of disulfide bonds with neighboring proteins. This point will be analyzed in detail in Section 4.3. Clear differences could also be noticed with respect to phosphate bands (cf. labels inset in Figure 7). In particular, the intensity ratio of the band at 1080 cm^−1^ (dibasic proton) to that at 979 cm^−1^ (monobasic proton) increased by a factor of ~1.7 in the OMV-exposed cells. A preponderance of –OPO_3_^2−^ bonds over –OPO_3_H^−^ bonds points to the depletion of hydrogen at the phosphate terminus, a trend similar to that observed above for the hydrogen polysulfide bonds. This point will also be further discussed in Section 4.3. Two doublets could also be resolved, namely, the one related to the tyrosine residues at ~825/850 cm^−1^ (i.e., arising from out-of-plane C−H bending and in-plane ring breathing, respectively) [96], and another one related to tryptophan at 1340/1361 cm^−1^ (i.e., arising from C−H bending and indole ring stretching, respectively) [97,98]. These two doublets, the relative intensities of which clearly differed between the OMV-exposed and control cells, are both quite sensitive to environmental pH and, as discussed further in Section 4.2, provide unique insight into the cells’ chemical environment. Additional bands belonging to tryptophan, which could be seen at 1550 and 1574 cm^−1^ (i.e., arising from C=C stretching modes in the pyrrole and benzene rings, respectively) [99,100], did not show significant variation in terms of their intensity ratio. Regarding the secondary structure of proteins, as reflected in the Amide I bands (cf. labels in inset to Figure 7), significant variations could be observed, with a nearly twofold increase in β-sheet fraction in the OMV-exposed cells, at equal expense to both α-helices and random coils. This feature can be interpreted as a clear vibrational fingerprint for Aβ formation. A quantitative comparison of protein secondary structure between OMV-exposed and control cells will be presented in Section 4.2.

**Figure 8:**
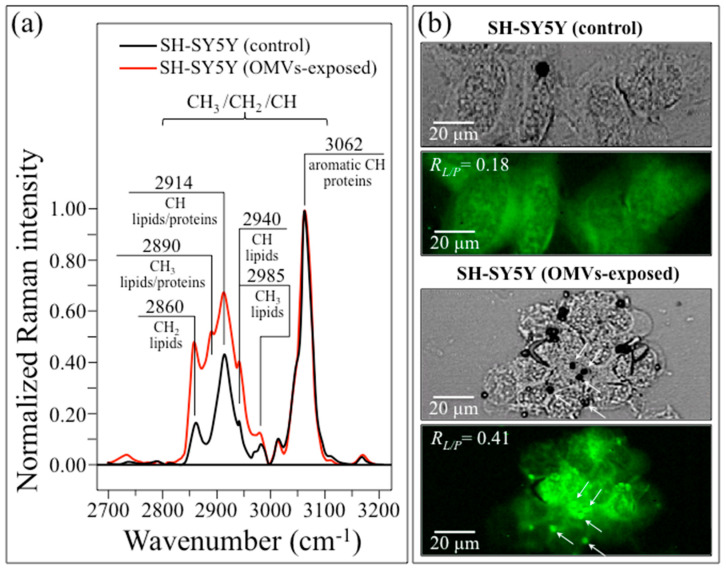
(**a**) Raman spectra in the high-frequency wavenumber interval 2700~3200 cm^−1^ after normalization to the band at 3062 cm^−1^ (aromatic C−H stretching in proteins; cf. all of the inset assignments) of control SH-SY5Y and Pg-OMV-exposed SH-SY5Y cell cultures. (**b**) Optical micrographs of control SH-SY5Y (upper) and OMV-exposed SH-SY5Y (lower) cell cultures with their respective Raman images for the intensity ratio RL/P = I2940/I3062 as a parameter for lipid concentration (average RL/P values are shown for each Raman map); the RL/P-intensified spots, which were only observed in the OMV-exposed culture (cf. arrows in inset), were interpreted as local accumulations of cholesterol.

Finally, the high-frequency wavenumber interval between 2700 and 3200 cm^−1^ was also investigated. Figure 8a shows spectra in this wavenumber zone after normalization to the band at 3062 cm^−1^ (aromatic C−H stretching in proteins; cf. the inset assignments) [101]. At high frequencies, the C−H bands from or contributed by lipids were all enhanced in relative intensity in the OMV-exposed culture. We selected the Raman intensity ratio R_L/P_ = I_2940_/I_3062_ as the parameter for lipid concentration and mapped this parameter for both the control and OMV-exposed SH-SY5Y cell cultures, as shown in Figure 8b,c, respectively. A comparison of these maps confirmed the enhancement of lipid concentration in the latter cell culture. Moreover, R_L/P_-intensified spots could also be clearly observed in the OMV-exposed cell culture (cf. the arrows in Figure 8c). We suggest that the presence of such spots of lipids could be conspicuously contributed by local accumulations of cholesterol. This interpretation will be substantiated in Section 3.3, below.

In conclusion, the present in situ Raman characterizations showed significant differences between OMV-exposed and control cell cultures. A detailed analysis of such differences could promote a spectroscopic interrogation of the molecular mechanisms underlying cell degradation, as well as Aβ and phosphorylated Tau neurotoxicity in the development of AD. Some aspects of those mechanisms are discussed Section 3.1, Section 3.2 and Section 3.3 below.

## 3. Discussion

### 3.1. Raman Insights into Pg-OMVs Molecular Structure and Functions

As demonstrated by previous studies on samples extracted from liver or joint fluid [37,102,103], Pg DNA can be found in remote parts of the human body even if Pg bacterial cells are not detected. A convincing explanation for this phenomenon was given by Nara et al. [19,104], who showed that Pg-OMVs could enter the brain through the blood–brain barrier rather than being carried by direct Pg infection. Pg-OMVs thus act as important carriers of Pg DNA and other signal molecules to distant organs without direct translocation of Pg bacterial cells [105]. Moreover, Pg-OMVs possess a higher bacterial virulence (i.e., 3~5-fold increase in gingipain levels) and increased antigenicity (resulting from higher concentrations of immune-responsive factors) compared to the respective levels detected at the surface of Pg bacterial cells [106,107,108]. Building upon this understanding, the present study confirmed that contamination by Pg-OMVs (in absence of Pg bacterial cells) suffices to induce severe AD-like degradation in human neuroblastoma cells. The present data also provide further evidence for the high virulence of Pg-OMVs.

Pg-OMVs contain several toxic substances, including lipopolysaccharides, fimbriae, and gingipains [109]. Pg-OMVs’ lipopolysaccharides are contaminated with PDHC lipids [110], a group of unusual and complex-structured lipids that account for the ability of Pg-OMVs to intensify inflammatory reactions, ultimately leading to destruction of tissue [111] and apoptosis of several cell types [112]. On the other hand, fimbriae and gingipains provide Pg-OMVs with adhesive and proteolytic abilities, respectively, with the former mediating adherence and entry into host cells, and the latter contributing destruction of cell structure and tissues [111]. PGDHC lipids can either include three aliphatic chains (i.e., the so-called “sub” form; cf. structure in inset to Figure 4a) or be de-esterified to two aliphatic chains (i.e., the so-called “unsubstituted”, or simply “un”, form) [110,113]. According to the results of high-performance liquid chromatography performed by Nichols et al. [39], SubPGDHC (including low- and high-mass ones) represent a minor (~25%) fraction of the total PDHC lipids found in diseased brain tissue. However, their pathogenic effect on cells was reported as being the most detrimental [114]. Recent results by Yamada et al. [115] indicated that only Pg-derived PGDHC upregulated secretion of soluble Aβ42 peptide, expression of amyloid precursor protein (APP), and hyperphosphorylation of Tau protein in cell cultures. On the other hand, Pg-LPS had little effect on Tau protein degradation. Nevertheless, both PGDHC and Pg-LPS contributed to accelerate senescence in SH-SY5Y cells.

The present Raman data offer new insights into PDHC- and LPS-induced cellular degradation, and provide chemical foundations in support of previous findings on the origin of Pg-OMVs-driven AD pathogenesis. The strongest band, located at ~987 cm^−1^ (PO_3_^2−^) in the spectrum of SubPGDHC (cf. Figure 4a), which is also seen with similar intensity in the Pg-OMVs one (cf. Figure 4c), represents a key signal unique to Pg-OMVs when compared to vesicles produced by different bacteria. Upon specific calibrations, this band, which arises from symmetric stretching vibrations of phosphate groups [45,50,78], might be regarded as a sensor for the concentration of Pg-OMVs. Note that the Raman spectrum of Pg-LPS (cf. Figure 4b) lacks signals from phosphate groups, which might give a rationale for its having little effect on Tau protein degradation. However, the Pg-LPS spectrum is full of signals that do not belong to its basic structure. This is because it traps (and strongly holds) several different toxins. As a matter of fact, the Pg-LPS spectrum in Figure 4b is dominated by signals related to oxysulfur anions [59,60,61], which are strongly reactive species capable of either oxidizing or reducing tissues and cellular compounds. Several such Raman signals still appeared strongly in the spectrum of phosphorylated Tau (cf. Figure 6c) and, to a lesser extent, of Aβ (cf. Figure 6b). However, only one prominent band at 855 cm^−1^ in the spectrum of Pg-OMV-exposed SH-SY5Y cells could unequivocally be assigned to O−O bonds in oxysulfur anions (cf. insets in Figure 7b). Strong signals related to arginine and lysine residues were also found in the spectra of both Pg-LPS and Pg-OMVs spectra (cf. Figure 4b,c, respectively). The origin of these signals is definitely related to the presence of the so-called Arg- and Lys-gingipain. Arginine-specific and lysine-specific types of proteases are implicated in a wide range of pathological and physiological activities undertaken by Pg [19,104], which include disruption of host defense mechanisms, dysregulation of various cellular functions, inhibition of interactions between host cells and the extracellular matrix, and degradation of hemoglobin for the acquisition of both peptides and heme [116,117]. While it is clear that gingipain toxins (cysteine proteases) including oxysulfur anions can very efficiently serve as tools for the above destructive actions [118], these signals were not unambiguously resolvable in the spectrum of cell cultures, because of overlapping with other strong signals (including those arising from aromatic ring structures, S-containing amino acids, and CH_2_/CH_3_ bonds) (cf. insets in Figure 7a,b). Thus, they could not easily serve as Raman markers for Pg-OMV contamination in cell cultures or tissues. However, the unique O−O signal at 855 cm^−1^ from oxysulfur anions, which unequivocally represents a substantial part of the destructive “equipment” of Pg-OMVs, pairs the PDHC lipid fingerprint at 987 cm^−1^, with its unique non-mammalian character [119], and could serve to assess Pg-OMV fractions once Pg-OMVs have been efficiently extracted from samples of human saliva through, for example, high-yield magnetic harvesting [120].

### 3.2. Spectroscopic Evidence for an Alkaline Shift in Diseased Cell Culture

**Figure 9:**
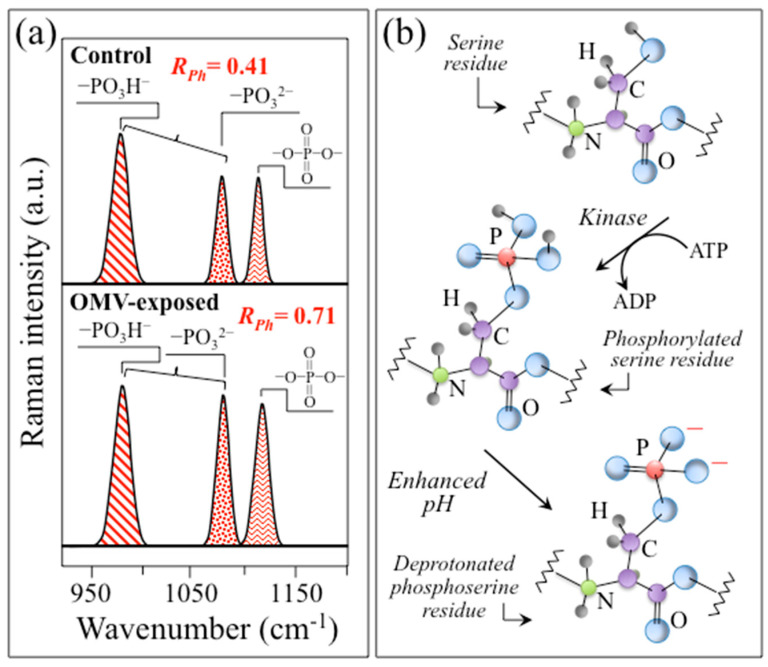
(**a**) Selected phosphate-related Raman signals as extracted from the spectra of the control SH-SY5Y (upper) and the Pg-OMV-exposed SH-SY5Y (lower) cell cultures shown in Figure 7a,b, respectively. Three characteristic stretching bands were selected and compared, which belong to: polyphosphates at ~1112 cm^−1^, dibasic phosphates at 1080 cm^−1^, and monobasic phosphates at 978 cm^−1^ (cf. the respective computed R_Ph_ = I_1080_/I_978_ ratios, inset); in (**b**), mechanism of relative fractional increase in–OPO_3_^2−^ and O=P=O bonds and their effect on serine residues.

Mandal et al. [121] reported and statistically validated data in which pH alterations were detected in patients with different brain disorders using magnetic resonance spectroscopy. A clear increase in intracellular pH was indicated in mapping hippocampal areas of AD patients, as compared with the unchanged pH in Parkinson’s patients. Two possible mechanisms have been suggested to explain the experimental observation of hippocampal alkalinization: (i) lipid peroxidation from oxidative stress; and (ii) oxidative post-translational modification. In brain cells, elevated levels of reactive oxygen species (ROS) lead to lipid peroxidation and to the formation of secondary intermediates, such as the 4-hydroxy-2-nonenal (HNE) aldehyde [122,123]. HNE is an extremely reactive species capable of forming protein adducts with a vast number of targets (i.e., preferentially, cysteine residues in thiol-containing proteins) [124,125]. In the case of AD, in addition to representing a biomarker of oxidative stress, HNE has also been shown to affect mitochondria by impairing adenosine triphosphatase (ATPase) activity [126]. Impairment as a result of increased HNE concentration occurs by inhibiting the synthesis of brain-type creatine kinase (BB-CK), namely, the enzyme that supports the regeneration of ATP from adenosine diphosphate (ADP), according to the following enzymatic reaction [127]:

(1) $$\mathrm{ATP}+\mathrm{Cr}\;\overset{BB-CK}{\to }\mathrm{ADP}+\mathrm{PCr}+H^{+}$$

where Cr and PCr represent creatine and phosphocreatine, respectively. The inhibition of BB-CK enzyme synthesis by HNE slows down Equation (1), and thus reduces H^+^ production (thus resulting in the observed pH increase). Oxidative posttranslational modification has been reported to induce a similar effect on BB-CK [128]. In support of clinical data, the present study consistently provides several independent proofs of pH enhancement in AD SH-SY5Y cells by means of in situ Raman spectroscopy. It should be noted that a direct confirmation of ATP redundancy in the Raman spectrum of diseased cells at the expense of ADP is difficult because of the overlap of several stronger signals from different molecules (cf. Figure 7). Nevertheless, a stronger signal in diseased cells at ~1112 cm^−1^ (cf. Figure 7b), which represents symmetric stretching in polyphosphates (i.e., involving combinations of O=P=O bonds) and is thus more pronounced in ATP than in ADP [129], definitely supports the hypothesis of a conspicuously dormant Equation (1). It should be noted that a fully protonated phosphate structure, –OPO_3_H_2_, is only stable under highly acidic conditions (i.e., pH < 2), while in the pH range 3~10, only monobasic and dibasic structures (i.e., –OPO_3_H^−^ and –OPO_3_^2−^, respectively) are present. The phosphate species –OPO_3_H^−^ and –OPO_3_^2−^ display characteristic Raman signals at 978 and 1080 cm^−1^, respectively [79], and their Raman intensity ratio, R_Ph_ = I_1080_/I_978_, could be used in local pH assessments. As previously presented in Section 2.3, Raman features associated with these two ionic forms were clearly distinguishable in the Raman spectrum of the phosphate groups present in both the control and diseased cell cultures. This approach is similar to that used in previous studies of free phosphate ions and nucleic-acid-linked phosphate groups [130,131,132]. A ~45% increase in the spectroscopic ratio, R_Ph_, which is representative of the dibasic-to-monobasic ratio, was observed for the diseased cell culture as compared to the control one. This spectroscopic difference is clearly visualized in Figure 9a by extracting selected phosphate bands from the spectra in Figure 7. This comparison gives a further hint for peptide phosphate protonation under AD conditions [79]. According to the calibrations shown in Ref. [79], the observed variation in R_Ph_ ratio in the diseased cell culture roughly corresponds to a pH enhancement of 0.2~0.4. It remains to be discussed how this difference in protonated termini affects AD progression from a molecular chemistry viewpoint. As a possible link between the environmental alkalinization and phosphorylation of selected amino acid residues, Figure 9b shows how a relative fractional increase in–OPO_3_^2−^ and O=P=O bonds could occur in serine residues. Protein phosphorylation represents a common chemical step in cell physiology [133]; however, as discussed in detail in the next section, abnormal phosphorylation (or hyperphosphorylation) triggered by alkalinization of the cell environment is often related to neurodegenerative disorders, including AD [134,135].

Additional spectral features in support of the thesis of an alkaline environmental shift in AD cell cultures as compared to control are represented by the spectroscopic behaviors of two characteristic doublet ratios: tyrosine (R_Tyr_ = I_850_/I_825_) and tryptophan (R_Trp_ = I_1361_/I_1340_). The tyrosine (or Fermi) doublet at 825 and 850 cm^−1^, which, as previously mentioned, arises from out-of-plane C−H bending and in-plane ring breathing, respectively, is quite sensitive to the surrounding environment and provides information on the cells’ chemical environment [96]. The intensity ratio, R_Tyr_, is diagnostic of the H-bonding environment around tyrosine residues, the lower the ratio, the more hydrophobic the tyrosine configuration. An increasingly alkaline environment increases tyrosine hydrophobicity, and vice versa for an acidic environment. Accordingly, increasingly low R_Tyr_ values are a sign of surface protonation and point to alkaline and more hydrophobic states of proteins.

**Figure 10:**
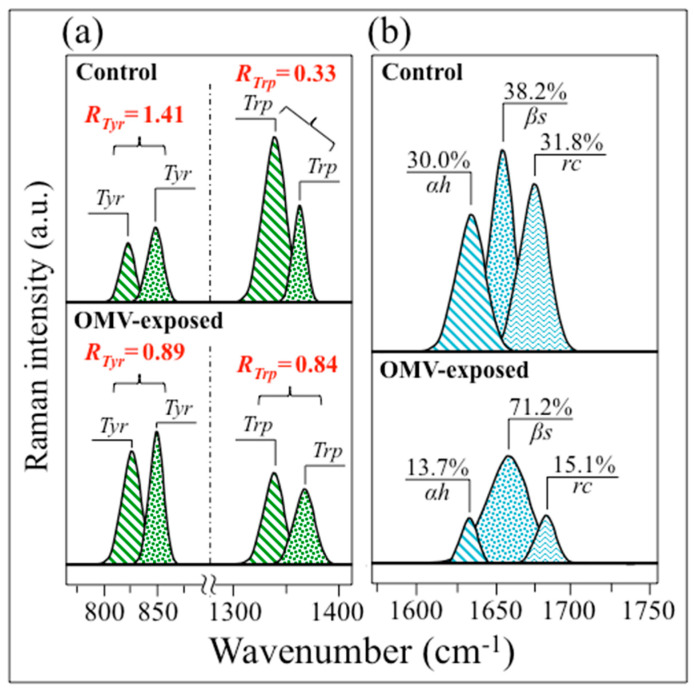
(**a**) Raman doublets belonging to tyrosine and tryptophan as extracted from the spectra of the control SH-SY5Y (upper) and the Pg-OMV-exposed SH-SY5Y (lower) cell cultures shown in Figure 7a,b, respectively. Computed tyrosine, R_Tyr_ = I_850_/I_825_, and tryptophan, R_Trp_ = I_1361_/I_1340_, ratios are given. (**b**) Amide I bands, which are characteristic of the secondary structure of proteins, are extracted from the spectra of the control SH-SY5Y (upper) and the Pg-OMV-exposed SH-SY5Y (lower) cell cultures shown in Figure 7a,b, respectively. The fractional amounts of different secondary structures as computed from areal fractions of the three characteristic Amide I signals are given. Abbreviations are the same as those described for Figure 6.

As seen in Figure 10a, AD cells showed a nearly twofold decrease in tyrosine ratio compared to control cells (R_Tyr_ = 0.89 vs. 1.41). The R_Tyr_ = 0.88 value measured for AD cells has been reported to be close to that of ionized molecules [96]. Accordingly, the Raman response of tyrosine residues proved a clear pH shift towards alkalinization. Also consistent with the tyrosine behavior is the response of the tryptophan doublet, whose signals at 1340 and 1361 cm^−1^ arise from C−H bending and indole ring stretching, respectively [97]. Similar to R_Tyr_, the tryptophan doublet ratio, R_Trp_, is also an indicator of hydrophobicity and thus links to environmental pH [97,98]. The 1361 and 1340 cm^−1^ bands are stronger in hydrophobic and hydrophilic environments, respectively. Figure 10a shows that AD cells exhibited a nearly threefold higher tyrosine ratio than the control cells (R_Trp_ = 0.84 vs. 0.33), thus proving again the alkaline shift in diseased cells. According to calibrations by Harada et al. [97], the observed change in R_Trp_ corresponds to a alkaline shift in pH of about 0.3 from physiological conditions (which is in reasonable agreement with the increase in pH determined from R_Ph_; cf. above).

Finally, the significant alteration in the protein secondary structure observed in diseased cells as compared to the control ones (Figure 10b) is also consistent with an increase in the pH of the cellular environment [136]. In diseased cells, a nearly twofold increase in β-sheets was accompanied by reductions of ~1/2 of the original fractions of both α-helices and random coils. The structure of insoluble Aβ plaques in AD cells has been reported to be rich in β-sheets. It should be noted that Aβ oligomers adopt a non-standard secondary structure (referred to as an “α-sheet”) [137], which forms in an early stage of aggregation. However, in a later stage, β-sheet fibrils are produced, and this is indeed the stage examined here; β-sheet-structured fibrils consist of tightly bound toxic oligomers and lead to neurotoxicity in neuroblastoma cells [137].

Regarding Tau structure, Avila et al. [84] and Guo et al. [138] described the molecular structure of Tau-paired helical filaments as being a precursor to plaque formation. However, their secondary structure remains controversial. Early X-ray diffraction studies pointed to a β-pleated sheet structure [86], while later studies suggested a structure devoid of any secondary patterns [139]. Due to their flexible and intrinsically disordered nature, Tau proteins might exhibit a large number of different conformations, which are difficult to resolve using crystallographic methods. The present vibrational data, however, provide clear structural information regarding the overall secondary structure of phosphorylated Tau peptides (cf. Figure 6d), although they do not make it possible to distinguish whether the clear increase in β-sheet structure observed in AD cells as compared to control cells mainly arises from Aβ plaques or Tau tangles.

In summary, several pieces of evidence in our Raman study suggest that an increase in environmental pH triggers a cascade of intermolecular interactions, which ultimately stabilize amyloid fibrils and Tau-paired filaments. These spectroscopic results are in agreement with previous studies describing the factors affecting the aggregation of amyloidogenic and tauogenic protein molecules, in which disulfide bonds and phosphate groups, respectively, play critical roles [140,141,142].

### 3.3. Insights into AD Human Neuroblastoma Cells from Molecular Chemistry

The progression of the neurodegenerative Alzheimer’s disorder is known to occur through the accumulation of toxic amyloid plaques and neurofibrillary tangles in the brain, accompanied by synapse and neuron loss [143]. Although it has been demonstrated that Aβ and Tau synergistically impair mitochondrial respiration in transgenic Alzheimer’s disease mice [134,144], the exact mechanism behind the impairment is yet to be clarified. In a separate experimental approach, extensive evidence was provided that mitochondria play a key role as a major source of oxidative stress in AD [145]. Mitochondria play an essential role in the functionality of neuronal cells, because these cells possess limited glycolytic capacity, and thus necessarily need to rely on aerobic oxidative phosphorylation for their energetic needs. However, oxidative phosphorylation is a major source of ROS such as hydrogen peroxide (H_2_O_2_), hydroxyl (•OH), and superoxide (O_2_^−^•), which are the products of regular cellular respiration [146]. Therefore, in the case of abnormal mitochondrial turnover (e.g., reduction in mitochondria and subsequent mitochondrial protein accumulation) and/or reduced cerebral metabolism, as in the case of AD [147], the reactive species hits cellular targets, i.e., lipids, proteins, and DNA, including mitochondrial components themselves [148]. As discussed in Section 2.4, the present in situ Raman study shows that oxidative modification of cysteine residues (i.e., presumably by oxygen peroxide, H_2_O_2_) yields an increase in Raman signals traceable to S–S bonds.

**Figure 11:**
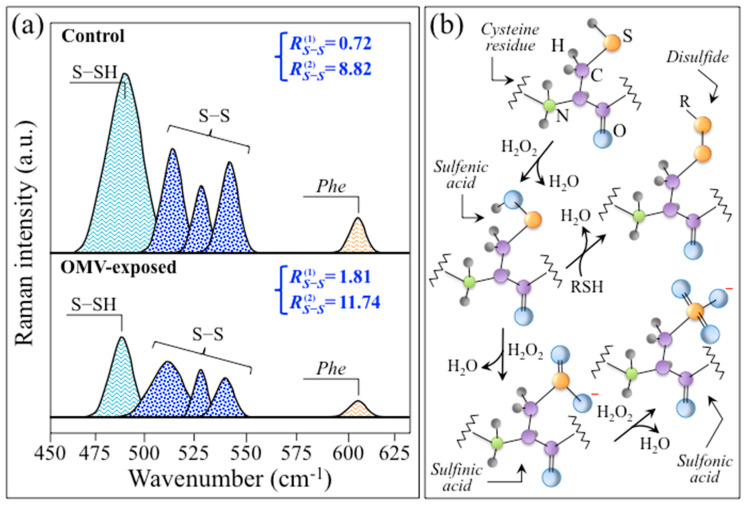
(**a**) Spectroscopic difference in S–S bond structures as observed upon the extraction of the selected Raman bands for control and AD SH-SY5Y cells (cf. upper and lower parts, respectively) from the spectra in Figure 7. Two different spectroscopic ratios were computed from the areas subtended by selected Raman signals: R^(1)^_S-S_ = (I_507_ + I_523_ + I_545_)/I_490_, which gives the sulfide-to-sulfhydryl bond ratio; and R^(2)^_S-S_ = (I_507_ + I_523_ + I_545_)/I_606_, which gives the variation in the sulfide bond population with respect to phenylalanine residues. Both parameters indicate a clear increase in the population of disulfide bonds in the diseased cell culture (cf. values in inset). (**b**) A schematic draft for the suggested mechanism of cysteine degradation by H_2_O_2_ radicals, which ultimately leads to the hyperoxidation of sulfenic acid to irreversibly form sulfinic and sulfonic acids as first proposed in Ref. [150].

Figure 11a clarifies the spectroscopic difference in S–S bond structures by extracting selected Raman bands from the spectra in Figure 7 from control and AD SH-SY5Y cells (upper and lower parts, respectively). This comparison might provide further chemical insight into AD progression in the cellular structure. In support of the detrimental effect of S–S bond structures in cysteine residues, a recent study by Saito et al. [149] showed that disulfide bonds formed in cysteines stabilize Tau proteins and contribute to Tau accumulation under oxidative stress. It should also be noted that the formation of sulfenic acid represents the first step in cysteine degradation (Figure 11b) [150]. However, this molecule might prove conspicuously unstable, depending upon its accessibility to additional thiols. The presence of a second cysteine residue in a neighboring protein might yield a disulfide bond (an alternative path is a reaction with glutathione that might lead to a specialized mixed disulfide, referred to as glutathione disulfide). Moreover, in the presence of excess H_2_O_2_, e.g., under conditions of oxidative stress, sulfenic acid undergoes hyperoxidation to irreversibly form sulfinic and sulfonic acid (cf. Figure 11b) [150]. The occurrence of oxidative modifications of cysteine residues might also explain why the KYT gingipain inhibitor fails to inhibit the formation of the Tau protein in the present cultures (cf. Figure 2c).

According to the above reasoning, we shall assume that in the present in vitro experiments, too, ROS could be responsible for specific oxidative modifications of proteins, thus altering their functionality. Specific consideration is then given to cysteine residues. While the majority of cytoplasmic proteins contain cysteine sulfhydryls that hardly react with ROS because of their high pK_a_ value (>8), some redox-sensitive proteins incorporate cysteine residues as thiolated anions even at neutral pH as a consequence of interactions with neighboring positively charged amino acids. In such redox-sensitive cysteine residues, thiolated anions can easily undergo oxidation to form sulfenic, sulfinic, and sulfonic acids, as well as protein disulfides (i.e., as shown in Figure 11b) [151,152]. Among the highly redox-sensitive molecules is glyceraldehyde-3-phosphate dehydrogenase (GAPDH), a tetrameric enzyme containing an essential cysteine sulfhydryl (referred to as Cys-149), which has long been known to be prone to oxidation or thiol-alkylation [153,154]. Among its many and diverse activities [155,156], it is now widely recognized that GAPDH plays a key proapoptotic role and participates in neuronal death in AD [157]. The deposition of insoluble extracellular Aβ-plaque deposits has been associated with elevated ROS levels; [158] these deposits contain GAPDH, which binds the carboxy-terminus of the APP to Aβ [159], and tend to aggregate, forming disulfide-linked multimers [160]. According to the general notion that the regulation of pH is essential for a correct neurophysiology [161], our in situ Raman experiments suggest that the observed increase in pH might be associated with GAPDH release in the extracellular space and the subsequent formation of insoluble disulfate linkages. Therefore, pH enhancement could be regarded as a trigger for the extracellular accumulation of disulfide-linked GAPDH, which in turn implies a chemical origin for the formation of insoluble Aβ plaque deposits as the main signatures of AD pathophysiology.

**Figure 12:**
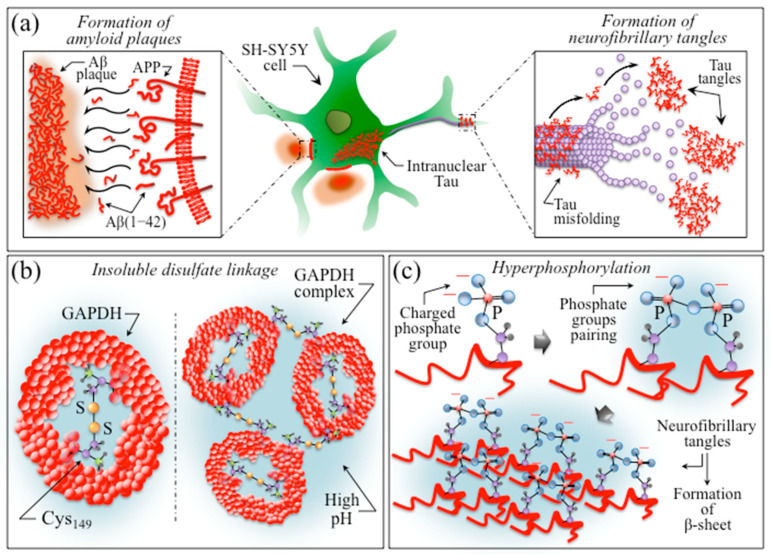
(**a**) Molecular processes involved in the formation of Aβ(1-42) plaques and neurofibrillary Tau tangles in the extracellular space (redrawn based on Refs. [143,163]); (**b**) schematic draft of the proposed mechanism for the formation of insoluble disulfate bonds at cysteine residues to form GAPDH complexes in increasingly alkaline environments; and (**c**) schematic draft of the mechanism of hyperphosphorylation by phosphate group pairing on serine residues triggered by the alkaline environment following exposure to creatine kinase BB-CK kinase.

In a recent paper, Pospich and Raunser [143] described the molecular process for the formation of Aβ(1-42) plaques [162] in extracellular space based on reconstructed cryo-electron microscopy images and solid-state nuclear magnetic resonance analyses performed by Gremer et al. [163] The process was represented as a sequence consisting of a cleavage step performed on transmembrane APP by β- and γ-secretase enzymes in neurons, followed by the formation of toxic oligomers and aggregates of amyloid fibrils [164]. A schematic draft of the proposed pathogenic process is presented in Figure 12 [143,163]. Figure 12b depicts the formation of disulfide-linked multimers and their chemical origin. Building upon the above notions regarding the role of GAPDH in AD, our in situ Raman screening of Pg-OMV-exposed SH-SY5Y cells suggests an enhanced presence of metabolic fingerprints for disulfide-linked multimers compared to the control culture. As previously mentioned in Section 2.3, the S–S triplet at 507, 523, and 545 cm^−1^ arises from different cysteine complex conformations [73,79], while the more intense band at 490 cm^−1^ can be ascribed to S–SH stretching [79,82]. It is expected that, in an increasingly alkaline cellular environment, the sulfane-to-sulfhydryl ratio of bond population will increase. A computation of the Raman spectroscopic ratio, R^(1)^_S-S_ = (I_507_ + I_523_ + I_545_)/I_490_, for the control and Pg-OMV-exposed SH-SY5Y cells does indeed show a ~2.5-fold increase in the latter cell culture (cf. the values inset in Figure 11a). An additional spectroscopic parameter showing the increase in the S–S bond population could be found with the ratio R^(2)^_S-S_ = (I_507_ + I_523_ + I_545_)/I_606_, where the disulfide triplet is normalized to the C–C=O in-plane bending signal from phenylalanine [69]. In this case, as well, a clear increase by ~25% is recorded (cf. the values inset in Figure 11a), which is consistent with the statistically higher population of disulfide bonds in the diseased cell culture. The multiple and reciprocally consistent Raman fingerprints pointing to an alkalinization of the AD cell environment (cf. Figure 9 and Figure 10, and the discussion in the previous section) give a rationale for the formation of disulfide bonds in redox-sensitive cysteine residues, as well as the appearance of enhanced signals contributed by O–O bonds at 855 cm^−1^ that are traceable to peroxydisulfate and thiosulfate ions (cf. Figure 7b) [165].

Following a previous study by Ledesma and Dotti [166], Chen et al. [167] indicated cholesterol as being one of the fundamental factors involved in both the generation and deposition of Aβ, and tracked it on AD cells using Raman spectroscopy. Bidirectional modulation of the β-amyloid precursor protein, which produces Aβ through cleavage (cf. Figure 12a), was shown to be mainly operated by cholesterol. Therefore, cholesterol should be considered as a hallmark in the histopathology of AD patients [168]. The morphologies of the Raman bands in the high wavenumber interval 2800~3100 cm^−1^, which represent C–H stretching vibrations (cf. Figure 8a), appear to confirm this thesis, with a clear increase in the relative intensity of all bands from or contributed by lipids. The ratio R_L/P_ = I_2940_/I_3062_ was selected, since the band at 2940 cm^−1^ (=CH_3_ symmetric stretching) is specifically representative for cholesterol and cholesteryl linoleate among lipids [169], while the band at 3062 cm^−1^ (=CH stretching in the ring of aromatic amino acids) can only be found in proteins [170]. Notably, cholesteryl linoleate represents the preferred molecular form for cholesterol transport and storage, and its accumulation has been shown to be associated with cardiovascular diseases [171,172]. The Raman maps in Figure 8b display spots of such lipid accumulation in OMV-exposed cells that were not observed in the control cell culture. Accordingly, the present data could be interpreted as indicating storage spots for excess cholesterol active in the production of Aβ from β-amyloid precursor protein, which is in agreement with the previously published literature [166,167,168,171,172].

The progressive deposition of misfolded hyperphosphorylated Tau is another pathological indicator of Alzheimer’s disease [173]. The molecular mechanisms governing the intercellular spread of Tau proteins have been studied by Katsinelos et al. [174]. According to their studies, phosphorylated Tau proteins first accumulate intracellularly, before being secreted by direct translocation across the plasma membrane to the extracellular space to form tangles (cf. Figure 12a). Increased secretion is favored by hyperphosphorylation, which triggers microtubule detachment, thus increasing the availability of free protein strands within the intracellular space. Free Tau strands then interact with specific molecules enriched at the inner side of the plasma membrane across which they are finally released upon mediation by sulfated proteoglycans. The property whereby free monomeric or oligomeric Tau proteins are able to bind to membrane lipids (e.g., phosphatidylcholine) is an intrinsic chemical property, while their extracellular aggregation necessitates the presence of highly negatively charged sulfated proteoglycans (i.e., the common method used for producing in vitro Tau aggregation) [175]. As suggested by several studies [176,177,178,179,180], the increase in intracellular phosphorylation in AD is linked to the dependence of receptor activation on the endogenous production of H_2_O_2_ radicals that target cysteine residues [150,151,152,181]. Redox-regulated serine/threonine kinases are activated by intermolecular disulfide formation between homodimers, which enhances its affinity to the target proteins [182,183]. The production of ROS could thus be considered the common chemical origin for both the formation of insoluble Aβ plaque deposits (cf. earlier in this section) and hyperphosphorylated Tau tangles. In order to correctly interpret the obtained results, it should be considered that Tau hyperphosphorylation is the direct result of an imbalance between redox-regulated protein kinases and phosphatases [184]. While Tau hyperphosphorylation does actually carry out a neuroprotective function, its prolonged occurrence leads to AD and other neuropathologies. Despite the wide range of investigations suggesting that the pattern of phosphorylation is key to determining Tau structure and aggregation, the proposed mechanisms leading to Tau hyperphosphorylation in AD pathogenesis still present several ambiguities and unclear aspects [185]. This study suggests a link between environmental alkalinization and an increase in the deprotonation of phosphorylated amino acid residues (cf. Equation (1)). This is based on the spectroscopic evidence of relative fractional increases of –OPO_3_^2−^ and O=P=O bonds with respect to the –OPO_3_H^−^ bond in AD cells as compared with control cells (cf. Figure 9a). As an example of possible reactions, Figure 9b presents a schematic overview of the phosphorylation of serine residues upon kinase reaction and the subsequent deprotonation of phosphoserine residues upon pH enhancement, which is in agreement with the Raman data recorded for AD neuroblastoma cells. We thus suggest that the spectroscopic ratio R_Ph_ = I_1080_/I_978_ could be directly related to the reorganization into Tau-paired phosphate groups of helical filaments of Tau proteins (which is in agreement with a recent hypothesis by Rani and Mallajosyula) [186]. Note, however, that Raman data cannot clarify which specific amino acid residue, among serine, threonine, and histidine, is taking the predominant role in dimer formation. The significant fractional increase in β-sheet secondary structure observed for the AD cell culture as compared to the control one (from 38.2 to 71.2%; cf. Figure 10b) is in agreement with the high-resolution cryo-electron microscopy observations reported by Fitzpatrick et al. [187] on Tau filaments extracted from AD human cerebral cortex. As mentioned in the previous section, an additional and more general confirmation is given by the fact that an alkaline environment is known to favor the formation of β-sheet secondary structure in proteins. A schematic draft summarizing the above description of the molecular processes behind Tau tangle formation is offered in Figure 12c.

In summary, the Raman spectrum of diseased SH-SY5Y cells provides evidence for: (i) the increased presence of disulfide-linked compounds, likely including GAPDH, thus identifying the chemical origin of the formation of insoluble Aβ plaque deposits; (ii) the formation of storage spots for excess cholesterol to trigger the production of Aβ from β-amyloid precursor protein; and (iii) an enhanced protein phosphorylation as a precursor event for the reorganization into Tau-paired phosphate groups of helical filaments in Tau proteins.

Overall, the present study confirms and gives a molecular-scale rationale to the pioneering findings of researchers at the School of Dentistry at the University of Central Lancashire in the UK, who first revealed the links between periodontal disease and Alzheimer’s disease [188,189].

## 4. Materials and Methods

### 4.1. Isolation and Characterization of Pg-OMVs

Pg-OMVs were prepared from *P. gingivalis* culture supernatant as described previously [38]. Briefly, *P. gingivalis* ATCC33277 was grown in BHI-broth supplemented with hemin and menadione under anaerobic conditions. Ammonium-sulfate-sedimented proteins in culture supernatant were collected by centrifugation and then dialyzed against 10 mM sodium phosphate buffer at pH 7.4. Protein concentration was then determined using the Protein Assay CBB Solution (Nacalai Tesque, Kyoto, Japan).

Transmission electron microscopy (TEM) observation was performed using a JEM-2100 (JEOL, Tokyo, Japan) with an acceleration voltage of 80 keV. The Pg-OMVs were negatively stained with 0.1% phosphotungstate. The dynamic light scattering (DLS) method was used for assessing the size distribution of Pg-OMVs using a commercially available particle size analyzer (ELSZ-1000, Otsuka Electronics, Osaka, Japan) at 25 °C. The light source was a He-Ne laser with a wavelength of 630 nm set at an angle of 45°. Experimental data were analyzed using the algorithm provided by the manufacturer.

### 4.2. Purification/Activation of Gingipains and Isolation of PDHC

Pg-OMVs concurrently carry many different proteins (enzyme gingipains and fimbrial proteins), which are derived from the bacterial outer and periplasmic space [37]. For this reason, they were purified and activated with cysteine according to the method previously described by Kariu et al. [38] The activated proteases were diluted with 0.1 M Tris-HCl (pH 7.6) buffer (Fujifilm Wako Pure Chemical Corporation, Osaka, Japan) containing 50 mM NaCl (Fujifilm Wako Pure Chemical Corporation) and 5 mM CaCl_2_ (Nacalai Tesque, Kyoto, Japan) directly before assays. Phosphorylated dihydroceramide was isolated as described in the previous literature [42]. Purity of the lipid isolate was confirmed by liquid chromatography–mass spectrometry, and its structure verified prior to Raman analyses by means of electrospray ionization (ESI) MS/MS (as described in Ref. [42]). For biological experiments, PGDHC was dissolved in 70% ethanol.

### 4.3. Amyloid β, Phosphorylated Tau, and Their Characterizations

Aβ1-42 was obtained from the Peptide Institute (Osaka, Japan). Fibril formation of Aβ1-42 (22 μM) was monitored upon measuring the fluorescence intensity of 20 μM thioflavin T (ThT; Sigma-Aldrich, St. Louis, MO, USA) in phosphate-buffered saline (PBS). The lyophilized sample of Aβ1-42 was first dissolved in 0.1% ammonia solution to prepare the stock solution. The stock solution of Aβ1-42 was centrifuged at 53,000 rpm at 4 °C for 3h using a Himac CS120GX ultracentrifuge (Hitachi Koki Co., Ltd., Tokyo, Japan) to eliminate the preformed aggregates. Then, 90 μL of PBS containing ThT in PBS was mixed with 10 μL of the Aβ1-42 stock solution. The fluorescence measurements were carried out using a Genios plate reader (TECAN, Männedorf, Switzerland) with an excitation wavelength of 450 nm and emission wavelength of 485 nm in a polystyrene 96-microwell plate. 4R-Tau (P301L) fibrils with molecular weight 16.0 kDa were purchased from Cosmo Bio Co., Ltd. (Tokyo, Japan).

### 4.4. Cell Culture and Immunochemistry Assays

Human neuroblastoma SH-SY5Y cells were pre-cultured in RPMI1640 (Nacalai Tesque, Kyoto, Japan) supplemented with 10% heat-inactivated fetal bovine serum and 1% penicillin-streptomycin (Nacalai Tesque, Kyoto, Japan) in a 5% CO_2_ incubator. Cells were seeded at a density of 4 × 10^5^ cells/mL in 6-well glass-bottom plates (MatTek Corporation, Ashland, MA, USA), manually coated with Recombinant Human Laminin-511 E8 Fragment iMatrix-511 (Matrixome Inc., Osaka, Japan), and then incubated in a CO_2_ incubator at 37 °C overnight. After changing the culture medium to a new serum-free medium containing 1% penicillin-streptomycin either without or with Pg-OMVs (concentration of 10 μg/mL), the cells were further incubated for 6 h for observation and analyses.

The Cell Count Reagent SF (WST-8; Nacalai Tesque, Kyoto, Japan) was used for colorimetric cell viability tests after 6 h cell exposure to OMVs. An additional SH-SY5Y cell sample was analyzed for comparison, which was combined with Pg-OMVs treated with an inhibitor for Arg- and Lys-gingipains (KYT-1; Peptide Institute, Inc., Ibaraki-shi, Osaka, Japan). Then, a water-soluble tetrazolium salt was applied to the culture supernatant and the absorbance at 490 nm was measured on a plate reader Infinite^®^ F50 (Tecan Group Ltd., Männedorf, Switzerland). Cells were fixed with 4% paraformaldehyde (PFA) for 10 min at room temperature (RT) and washed with 0.1% triton X in phosphate-buffered saline (PBS); then, they were used for fluorescence microscopy experiments. Cell staining was performed using primary antibodies: Anti-Phosphorylated Tau (Clone: C5) and Anti-Human Amyloid β (1-42) antibodies (Immuno-Biological Laboratories Co, Ltd., Fujioka, Gunma, Japan) for 60 min at room temperature. After washing with wash-buffer, cells were incubated with an Alexa 594 Goat Anti-Mouse IgG or Alexa 594 Goat Anti-Rabbit IgG (Thermo Fisher Scientific, Waltham, MA, USA), Hoechst 33342 (Dojindo, Co., Ltd., Kumamoto, Japan) and Phalloidin Alexa 488 (Thermo Fisher Scientific, Waltham, MA, USA) for 60 min at room temperature in the dark. The stained cell cultures were then observed under a fluorescence microscope (BZX710; Keyence, Osaka, Japan).

### 4.5. In Situ Raman Spectroscopy and Raman Imaging

In situ Raman experiments were conducted by means of a specially designed spectrometer (LabRAM HR800, Horiba/Jobin-Yvon, Kyoto, Japan) set in confocal mode. This equipment employs a holographic notch filter to concurrently provide high-efficiency and high-resolution spectral acquisitions. The wavelength of the incoming laser was 785 nm with the laser source operating with a laser power of 70 mW. The spectral resolution of ~1 cm^−1^ was achieved upon analyzing the Raman scattered light by a double monochromator connected with an air-cooled charge-coupled device (CCD) detector (Andor DV420-OE322; 1024 × 256 pixels); the grating used in the spectrometer had a resolution of 1800 gr/mm. The acquisition time of a single spectrum was typically 10 s. Three consecutive acquisitions were made at the same spot to minimize noise. The laser spot was ~2 μm when focused on the sample through a 50× optical lens. In collecting average spectra, sets of ten spectra were collected at different locations over areas of ~2 mm^2^. The raw Raman spectra were first subjected to a baseline subtraction procedure as preliminary optimized and standardized according to the asymmetric least square method [190]. After baseline subtraction, average spectra were deconvoluted into series of Lorentzian–Gaussian sub-bands using commercial software (LabSpec 4.02, Horiba/Jobin-Yvon, Kyoto, Japan). In performing this deconvolutional procedure, a machine-learning approach was applied, which employed an in-house-built automatic solver described in previous studies [29,191]. Briefly, an automatic solver, *S_av_*(ν), was employed, which exploits a linear polynomial expression of Lorentzian–Gaussian functions, *V*(Δν, σ, γ); where ν, Δν, σ, and γ represent the Raman wavenumber, the wavenumber shift from each sub-band maximum (ν_0_), the standard deviation of each Lorentzian–Gaussian component, and the full-width at half-maximum of the Lorentzian component, respectively. Solving for the minimum value of the algorithm given by the equation below was pursued to match the experimental data:

*S_av_*(ν) − Σ*_i_ α_i_*Σ*_j_ β_ij_V_ij_*(ν_0_, Δν, σ, γ) ≅ 0 (2)

where the index *i* locates each compound in a series of *n* compounds contributing to the overall spectrum, and the index *j* locates each Lorentzian–Gaussian sub-band of a series of *m* compounds in the Raman spectrum of each compound of an *n* series. A series of Lorentzian–Gaussian sub-bands was pre-selected from a library of compounds, which included lipids, triphosphates, polysaccharides, and other key biomolecules for a total of more than 40 different biomolecules. The pre-selection was made according to the chemical and structural peculiarities of the sample studied. The algorithm pinpointed the closest matches to the experimental spectra according to the following criteria: (i) preserving relative intensities (*β_ij_*), (ii) assigning spectral positions (ν_0_), and full-width at half-maximum (σ and γ) values for specific sub-bands from each elementary compound within ±3 cm^−1^. This latter value was set upon considering both spectrometer resolution and possible minor alterations of the molecular structure). The conditions imposed on band positions, relative intensity, and bandwidths provided the required mathematical constraints to univocally deconvolute the experimental spectra. When the solver located sub-bands that did not match with any from the chosen compounds, a search for an additional compound was launched in the library to match the unknown bands according to the same criteria as above. According to this computational work, spectra could be screened automatically for an appropriate deconvolution with the closest matching to the experimental spectrum, and sub-bands having primarily single-reference-molecule sourced signal intensity (>90%) could be isolated.

Raman imaging was performed by means of a dedicated Raman device (RAMANtouch, Nanophoton Co., Minoo, Osaka, Japan) operating in microscopic measurement mode (50× lens; numerical aperture, NA = 0.9) with confocal imaging capability in two dimensions. This Raman spectroscope achieved high in-plane spatial resolution (i.e., 300 nm) upon exploiting a specially designed spectrograph with completely compensated aberration. Specially designed confocal optics also allowed for high spatial resolution (~670 nm) along the out-of-plane z-direction. This Raman microscope was also capable of ultra-fast simultaneous acquisition of up to 400 spectra, greatly reducing laser irradiation time and thus enabling compatibility of the Raman scanning with cell life. The excitation source was a 532 nm solid-state laser operating with a power of 10 mW at the sample surface; a 300 grating was used, which led to a spectral resolution of ~2 cm^−1^ (spectral pixel resolution of 0.3 cm^−1^/pixel) with an accuracy in laser spot spatial location of 100 nm. Raman hyperspectral images were generated by means of commercially available software (Raman Viewer, Nanophoton Co., Minoo, Osaka, Japan). Lateral displacements involved steps of 500 nm for the laser focal point on the samples.

### 4.6. Statistical Analyses

OD analyses are presented as a mean value ± standard deviation of three independent experiments (*n* = 3). The statistical difference between cells exposed to *Pg*-OMVs and unexposed ones (control sample) was analyzed by Tukey–Kramer multiple comparison test. Values *p* < 0.01 were considered statistically significant and are labeled with two asterisks.

## 5. Conclusions

The action of *Pg* periodontal pathogen is known to extend far beyond the oral cavity, with *Pg*-OMVs being the carriers of its DNA and other virulent molecules towards distant organs without direct translocation of *Pg* bacterial cells. The present paper confirmed this view through in vitro experiments showing that the presence alone of *Pg*-OMVs in neuroblastoma cell culture sufficed to induce cellular degradation through the formation of Aβ plaques and phosphorylated Tau tangles. In a systematic spectroscopic approach, we first characterized the Raman signals of isolated *Pg*-OMVs with different signals from SubPGDHC and *Pg*-LPS, and found that the latter were contaminated by gingipains; then, we further extended the Raman approach to characterize isolated Aβ fibrils and phosphorylated Tau peptides. These preliminary assessments enabled us to locate Raman fingerprints of *Pg*-OMVs that could be used in future quantitative assessments of their concentration in human fluids and tissues. As a final step, we monitored in situ the Raman response of a living SH-SY5Y cell culture exposed to *Pg*-OMVs in comparison with an unexposed control one. This comparison, which also included Raman imaging, provided several pieces of reciprocally consistent spectroscopic evidence supporting increased environmental pH as being a main trigger for intermolecular interactions that ultimately stabilized amyloid fibrils and Tau-paired filaments. AD SH-SY5Y cells showed a clear increase in disulfide-linked compounds, likely including GAPDH, as the chemical origin of insoluble Aβ plaques, the formation of storage-like spots of cholesterol that trigger the production of Aβ from the β-amyloid precursor protein, and an enhanced protein phosphorylation prompting the reorganization of Tau helical filaments into Tau-paired phosphate groups.

In conclusion, the present Raman study marks some progress in understanding the chemical mechanisms behind AD progression under the effect of *Pg*-OMVs; it also further establishes, along the same lines as analytical studies by other authors, Raman spectroscopy as a powerful method for characterizing *Pg*-OMVs in their key molecular components, thus paving the way to fast and quantitative measurements of their concentrations in human saliva for preventive periodontitis assessments and early AD diagnostics.

## Data Availability

Data available on request.
